# Decreased Expression of CDC25A in Azoospermia as the Etiology of Spermatogenesis Failure

**Published:** 2018

**Authors:** Dwi Anita Suryandari, Yurnadi Hanafi Midoen, Luluk Yunaini, Sari Setyaningsih, Hans-Joachim Freisleben

**Affiliations:** 1- Department of Medical Biology, Faculty of Medicine, Universitas Indonesia, Jakarta Pusat, Indonesia; 2- Master Program Biomedical Sciences, Faculty of Medicine, Universitas Indonesia, Jakarta Pusat, Indonesia; 3- Medical Research Unit, Faculty of Medicine, Universitas Indonesia, Jakarta Pusat, Indonesia

**Keywords:** CDC25A, Infertility, Johnsen scoring, Spermatogenic failure

## Abstract

**Background:**

Spermatogenesis is a tightly regulated developmental process of male germ cells. The stages in spermatogenesis are mitosis, meiosis and spermiogenesis. One of the genes playing a role in meiosis is Cell Division Cycle 25A (CDC25A). Decreased expression of CDC25A is associated with failure of spermatogenesis and sperm retrieval. Infertility examination for azoospermia has been limited on histological examination. Hence, molecular research to find marker genes for infertility will improve the examination of testis biopsies.

**Methods:**

This research is a cross sectional study of 50 testicular biopsies with Johnsen scoring categories from scoring 2 to 8. Analysis of mRNA expression used qPCR and protein expression using immunohistochemistry. Statistical analysis with Spearman correlation was considered significant at p<0.05.

**Results:**

The result showed that transcript level and protein expression of CDC25A decreased in score 5 of Johnsen scoring categories. Moderate Spearman rho correlation (r=0.546) between mRNA relative expression and protein expression of CDC25A was significant at p<0.01.

**Conclusion:**

Decreased expression of CDC25A is associated with meiotic arrest as the etiology of spermatogenic failure in many azoospermic men.

## Introduction

Infertility is still a problem in reproductive health affecting about 10%–15% of couples (1). Infertility is defined as the inability of married couples to obtain offspring after a year of sexual activity without the use of contraceptives (2). Anatomical abnormalities, endocrine, immunological problems, failure of ejaculation and environmental exposure are factors that may cause infertility (3). Infertility with unknown causes is called idiopathic accounting for about 40% of all infertility cases in men (4). The reason for such high rate of idiopathic infertility is certainly due to the possibility of lack of understanding of the basic mechanisms in the regulation of the genetic control of fertility. About 15%–30% of infertility cases are related to genetic abnormality (5).

The European Guidelines on male infertility recommend Johnsen’s scoring for the assessment of the stage of spermatogenesis in the seminiferous tubules (6). Johnsen’s scoring comprises 10 categories; a value of 10 denotes complete and regular spermatogenesis with multiple spermatozoa and normal seminiferous tubular epithelium. The scoring categorizes the stages of impaired spermatogenesis gradually down to value of 1 with no seminiferous epithelium.

Disturbances of the process of spermatogenesis involve many genes, approximately 2000 genes are estimated to contribute to the process of spermatozoa formation (7). One important stage in spermatogenesis is the meiotic division that occurs when the primary spermatocyte develops into spermatid. Meiosis is the process of cell division through double cleavage and the reduction of the number of chromosomes. The regulation of meiosis at molecular level is not yet fully understood (8).

Several candidate genes have been identified in the regulation of meiosis in animal models. Cessation of spermatogenesis during meiosis (meiotic arrest) is one of the causes of infertility. Meiotic arrest of germinal cells is characterized by being able to enter the stages of meiosis but failing to continue the division process. Hence, seminiferous tubules only contain spermatocytes at the end of the development of germinal cells. Spermatocytes in meiotic arrest will accumulate in the tubules and degenerate. One of the regulator genes of meiotic progression is cell division cycle 25A (CDC25A) (8).

CDC25A gene produces a group of proteins that play a role in activating cyclin-dependent kinase (CDK) complex for the regulation of cell division cycles. CDC25A gene is located on chromosome 3 short arm region 21 (3q21) (8). In mammals, there are three isoforms of CDC25, CDC25A, CDC25B, and CDC25C (9). CDC25A isoform is expressed as the most dominant gene in testicles and plays an important role in spermatogenesis (8), particularly in the activation of CDK2-cyclin E and CDK2-cyclin A during the phase of Gap1/ synthesis (G1/S) and possibly also in Gap2/meiotic (G2/M) phase through activation of CDK1-cyclin B complex for the initiation of chromosomal condensation. Infertility tests with testicular biopsies have generally been limited to microscopic examination by providing an assessment to the process of spermatogenesis in each seminiferous tubule. Research on genes involved in spermatogenesis failure is important to improve the quality of testicular biopsy examination for the causes of infertility.

## Methods

### Subjects and samples:

This descriptive research with cross-sectional design was conducted in the Molecular Biology Laboratory of the Faculty of Medicine University of Indonesia and the Stem Cell Laboratory of Prof. Dr. Oemiyati in the Center for Biomedical and Health Technology Agency for Health Research and Development. This study research was conducted for six months from June to November 2015. Ethical clearance was obtained from Committee of the Medical Research Ethics of the Faculty of Medicine, Universitas Indonesia under No.853/UN2.F1/ETIK/2015. Research samples were 50 testicular biopsies classified based on Johnsen histology categories (6, 10). The biopsies were drawn in the Department of Urology, Dr. Cipto Mangunkusumo Hospital, Faculty of Medicine, Universitas Indonesia, where the informed consent was signed by all participants and immediately transferred to the Department of Medical Biology for further investigation.

### Testicular preparation (11):

For fixation, testicular tissue was soaked in formalin 10% for 24 *hr*. Two hours after fixation, the samples were cut cross-wise into two parts with a razor blade. To remove water from the tissues, samples were immersed in ethanol 70%, 80%, 95%, and 100%, for 30 *min* each.

In the clearing process, alcohol was removed from the tissue samples and replaced with a solution of benzyl benzoate for one day, followed by benzyl alcohol for 15 *min*. For embedding, the clearing agent was removed from the tissue and replaced with liquid paraffin at 60°*C* for about 3 *hr* to harden. The paraffinated samples were stored at 4–8°*C* in the refrigerator until used for sectioning.

The paraffin blocks were cut by means of a rotating microtome with a thickness of 5 *μm* in transverse direction. The cross section of the paraffin block was fixed to a glass object slide with Mayer’s albumin and then placed on a hot plate.

### Sample staining was done with hematoxylin-eosin (HE) after sample deparaffinization:

xylene, changed twice, every 5 minutes, followed by ethanol 100% (5 *min*), ethanol 80% (5 *min*), ethanol 70% (5 *min*), distilled water (1 *min*), hematoxylin (1–2 *min*), ethanol 70% (2 *min*), eosin Y solution (30 *s*), ethanol 95% (2 *min*), ethanol 100% (5 *min*), and xylene (2 *min*). The slides with dehydrated samples were mounted with Canada Balsam (5 *min*) and a cover glass for observation under the microscope.

### Deparaffinization and RNA isolation:

The detailed procedure of RNA isolation was based on the protocol of High Pure RNA Paraffin Kit (Roche, Germany). In brief, tissue samples from testicular biopsies in the form of paraffin blocks were placed on a bath containing a xylene solution, for 10 *min*. Then, absolute ethanol was transferred to the bath and incubated for 10 *min*. The preparation was crushed using a scalpel and moved into Eppendorf cups. The tissue was dried for 10 *min* at 55°*C* for RNA isolation (12).

Into the cups, 100 *μl* tissue lysis buffer was added, and 16 *μl* of 10% sodium dodecyl sulphate (SDS) and 40 *μl* of proteinase K working solution were homogenized by Vortex for a few seconds and incubated overnight at 55°*C*. Subsequently, 325 *μl* of binding buffer and 325 *μl* of absolute ethanol were admixed to the lysates and centrifuged at a speed of 8000 rotation per min (*rpm*) for 1 *min* in a combined high pure filter and collection tube. The obtained supernatant was removed (12).

Then, 500 *μl* of wash buffer I were added to the tube and centrifuged for 2 *min* at a speed of 8000 *rpm*. The supernatant obtained in the bottom collection tube was removed and 500 *μl* of wash buffer II were added, centrifuged for 2 *min* at 8000 *rpm*, and the supernatant obtained in the collection tube was removed. Again, wash buffer II (300 *μl*) were added, centrifuged for 2 *min* at 8000 *rpm*, and the supernatant in the collection tube was removed. The high pure filter tube was centrifuged for 2 *min* at 12000 *rpm* for drying and then placed into a new Eppendorf cup. Ninety *μl* of elution buffer were added and centrifuged for 2 *min* at a speed of 8000 *rpm* (12).

Thereafter, 10 *μl* of DNase incubation buffer and 1 *μl* of DNase I working solution were admixed to the eluate and incubated for 45 *min* at 37°*C*. Subsequently, 20 *μl* of tissue lysis buffer, 18 *μl* of 10% SDS and 40 *μl* of proteinase K working solution were added, homogenized by Vortex and incubated for 1 *hr* at 55°*C*. After incubation, 325 *μl* of binding buffer and 325 *μl* of absolute ethanol were admixed into a new combined high pure filter collection tube and centrifuged for 2 *min* at 8000 *rpm*. The supernatant obtained in the collection tube was removed and centrifugation repeated at 12000 *rpm* for drying (12).

Now, 500 *μl* of wash buffer I working solution were added to the reservoir, centrifuged for 2 *min* at 8000 *rpm*, and the supernatant obtained in the collection tube was removed. Then, 500 *μl* of wash buffer II working solution were added and centrifuged for 2 *min* at 8000 *rpm*, and the supernatant in the collection tube was removed. The high pure filter tube was centrifuged for 2 *min* at 12000 *rpm*, placed into a new 1.5 *ml* cup; 50 *μl* elution buffer were added and incubated for 2 *min* at 20°*C*. After incubation, the cup was centrifuged for 2 *min* at 8000 *rpm* to collect RNA, which was subsequently checked for concentration and purity, prior to cDNA synthesis (12).

### cDNA synthesis:

For cDNA synthesis, 10 *μl* of RNA, 1 *μl* of oligo (dt) primer, and 2 *μl* of ddH_2_O were incubated for 10 *min* at 65*°C* in a thermal block. Immediately after, the cups were cooled on ice. Into the cups containing the template-primer mix, the remaining components of the RT Mix Transcriptor RT Reaction Buffer, Protector RNase Inhibitor, dNTP and Transcriptor RT were added. Total reaction volume of 20 *μl* was incubated for 30 *min* at 55*°C* and then the transcriptor reverse transcriptase was inactivated by heating to 85*°C* for 5 *min* (13).

### Gene amplification with qPCR:

Relative expression analysis of CDC25A mRNA used quantitative real-time PCR (qRT-PCR). The primary sequences for the CDC25A gene were forward (F) 5′-CTACTGATGGCAAGCGTGTC-3′ and reverse (R) 5′-TCTCTCTCCACATACCGGCAC-3′.

Glyceraldehyde-3-phosphate dehydrogenase (GA PDH) gene was used as an external standard with forward sequence of 5′-GAAATCCCATCACCA TCTTCCAGG-3′ and reverse sequence of 5′-GA GCCCCAGCCTTCTCCATG-3′. The primers were designed to produce a PCR product of 87 base pairs (*bp*) that would amplify DNA from the base region of 1620 to 1706 (14, 15).

Quantitative analysis of gene expression was assessed by the method Livak (16). To obtain efficiency value and cycle threshold (Ct), amplification with quantitative real-time (qRT) PCR expression was performed using the SYBR Green I No-Rox kit (Bioline) starting with a singleplex reaction of two replicates (duplo) with a total volume of 20 *μl* each, in a 96-well optical reaction plate. The qRT-PCR reaction mixture consisted of 5.4 *μl* of nuclease-free water, 0.8 *μl* of forward primer, 0.8 *μl* of reverse primer, 10 *μl* of SYBR I master mix and 3 *μl* of cDNA template with a concentration of 100 *ng/μl*. All qPCR components were incorporated into a 96-well reaction plate with an optical adhesive cover. No template control (NTC) was used to determine the occurrence of contamination during mixing of reagents qRT-PCR. The amplification process was initiated with 1 denaturation cycle at 95*°C* for 3 *min*, followed by a cycle at 95*°C* for 3 *s*, followed by 40 repeat cycles, annealing and elongation at 56*°C* for 30 *s* (16–19). For qRT-PCR, a 7500 Fast Real-Time Machine (Applied Biosystem, Foster City, United States) was used.

The cDNA synthesis results were further amplified and confirmed by electrophoresis in a 1.5% agarose gel at 90 volts for 42 *min*. The band of 87 *bp* indicated that the samples had good amplification efficiency. Thus, it was possible to continue with primer optimization using qPCR to find the primer attaching to the target gene.

### Immunohistochemistry:

Immunohistochemical staining used the TrekAvidin-HRP (horseradish peroxidase) complex labelling with the protocol and kit from Starr Trek Universal HRP-Detection System–Biocare Medical. The primary antibodies against CDC25A were mouse monoclonal antibodies. Positive CDC25A expression was represented by brown staining in the cytoplasm of spermatogenic cells. The microscopic assessment of all expression tests was done on the entire area of small field (magnification 100X) and large field (magnification 400X) (20).

The Johnsen scoring system for testicular biopsies categorizes value 10 for complete and regular spermatogenesis with multiple spermatozoa and normal seminiferous tubular epithelium. Category 9 denotes many end-stage spermatids, value 8 less than five spermatozoa and few late-stage spermatids. Value 7 means no spermatozoa, no end-stage spermatids, and many early-stage spermatids, whereas category 6 shows no spermatozoa, no end-stage spermatids, and only few early-stage spermatids. Score 5 denotes no spermatozoa or spermatids and many spermatocytes; score 4 no spermatozoa or spermatids and few spermatocytes. Score 3 shows only spermatogonia; score 2 no germinal cells, only Sertoli cells and in score 1 there is no seminiferous epithelium (6, 10). In our study, Johnsen score 8 was used as the control.

### Statistical analysis:

Statistical analysis used SPSS 22.0 software for Spearman correlation (21, 22).

## Results

### Molecular analysis:

RNA was isolated from testicular tissue samples of 50 infertile men with azoospermia at an average amount of 14.81 *ng/μl* and an average purity value of 2.02. Purity values of 1.9–2.1 indicate that the isolated RNA is pure (23). The subsequent synthesis of cDNA for the gene expression test of CDC25A had an average yield of 1481.13 *ng/μl* and a purity value of 2.17. Agarose gel electrophoresis showed a band of 87 *bp* indicating sufficiently good amplification efficiency and primer optimization could be continued using qPCR to determine if the primer binds correctly to the target gene.

The amplification results with the qPCR technique were described in the form of amplification curves. The target gene amplification curve of CDC25A shows the number of cycles with fluorescent signals correlated with the number of target cDNA initiations during the exponential phase of PCR. The amplification curve had a sigmoid shape and showed the value of the Ct indicating the number of cycles captured by the fluorescent signals which correlates to the amplification number (number of copies). The value of Ct varied between 21–29 with an average value of 26.245. In addition, CDC25 gene expression was calculated by comparison with the endogenous control GAP DH (16).

### Relative expression of CDC25A gene mRNA categorized by Johnsen scoring:

Six Johnsen scoring categories (groups 2 through 7) were determined *vs*. control (group 8=1); median values are presented in table 1. Median value in scoring 3 and 4 indicates an increase in the ratio of relative mRNA expression, but the expression declines in score 5 (0.7414) and then increases again in scores 6 and 7 (1.0974 and 1.0988, respectively), even slightly higher than controls. Statistical evaluation showed abnormal data distribution and significance (p< 0.05) of the correlation between CDC25A mRNA expression and Johnsen scoring.

**Table 1.** Expression of CDC25A mRNA based on Johnsen categories

**Johnsen categories**	**N**	**Median (min–max)**	**p**
**Scoring 2**	5	0.1365 (0.0007–0.7516)	<0.05
**Scoring 3**	5	0.8972 (0.0402–1.40073)	
**Scoring 4**	6	0.9261 (0.0390–1.6800)	
**Scoring 5**	10	0.7414 (0.03290–1.6838)	
**Scoring 6**	7	1.0974 (0.0390–1.9445)	
**Scoring 7**	11	1.0988 (0.0594–2.9644)	
**Scoring 8**	6	1 (control)	

Note: Scoring 8 (control) was set to 1; p=Significance of correlation between mRNA expression and Johnsen scoring

### Percentage of CDC25A positive cells based on Johnsen categories:

Immunohistochemical examination was carried out to determine CDC25A protein expression and visualize localization in spermatogenic cells during spermatogenesis. In table 2, the median percentages of positive cells in different Johnsen assessment categories are given. There is an increase from score 2 to 3 and 4, but in score 5, CDC25A protein expression declines and slightly increases again in score 6. Score 5 of Johnsen categories is characterized by primary spermatocytes that will enter meiosis. In scoring 7 and 8, the levels of mRNA expression are considerably higher than in all other groups. A general increase in the values can be seen from score 2 through 8, except for score 5, which decreases versus score 4. Score 6 slightly increases again although its value is still lower than score 4. Statistical evaluation showed strong positive Spearman rho correlation (r_s_=0.790) between Johnsen scoring and CDC25A-positive cells which was highly significant at p<0.001.

**Table 2.** Percentage of CDC25A-positive cells based on Johnsen categories

**Johnsen categories**	**N**	**Median (min–max)**	**p**
**Scoring 2**	5	13.36 (8.38–14.68)	<0.001
**Scoring 3**	5	34.79 (34.56–52.05)	
**Scoring 4**	6	46.45 (36.14–54.95)	
**Scoring 5**	10	38.24 (19.60–47.49)	
**Scoring 6**	7	41.23 (33.19–45.11)	
**Scoring 7**	11	66.97 (66.60–69.73)	
**Scoring 8**	6	84.11 (81.50–87.44)	

Note: CDC25A protein expression determined as CDC25A-positive cells; p=significance of correlation between CDC25A protein expression and Johnsen scoring

### Microphotograph of CDC25A Immunohistochemistry in testicular tissue:

**Figure 1.**
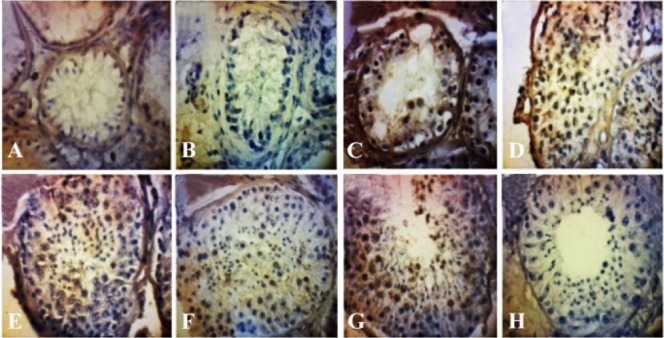
Microphotograph of immunohistochemical CDC25A staining in testis tissue (400X magnification). A: Johnsen scoring 2; B: Johnsen scoring 3; C: Johnsen scoring 4; D: Johnsen scoring 5; E: Johnsen scoring 6; F: Johnsen scoring 7; G: Johnsen scoring 8; H: negative control

Microphotographs with 400 X magnification show the process of spermatogenesis. CDC25A-positive cells are indicated by brown color of the cytoplasm. Score 2 is characterized by Sertoli cells only, scoring 3 by spermatogonium, scoring 4 is characterized by low amounts of primary spermatocytes, whereas scoring 5 is dominated by primary spermatocytes. Scoring 6 is characterized by the development of spermatogenic cells up to early spermatids and scoring 7 by the development of spermatogenic cells up to late spermatids. Scoring 8 is characterized by complete spermatogenesis from spermatogonium to spermatozoa. The negative control in figure 1H was prepared without primary antibodies, visible are only the blue nuclei of the spermatogenic cells due to counterstaining with hematoxylin Mayer.

### Correlation between mRNA relative expression and protein expression:

The correlation test between CDC25A mRNA expression and the number of CDC25A protein-positive cells showed moderate Spearman rho correlation (r_S_=0.546) with significance at p<0.01. In immunohistochemistry samples, 5 tubules for each sample were used to calculate the number of cells, which was compared with the mRNA expression values according to the type of sample in the immunohistochemical test.

## Discussion

This study examined the levels of mRNA and protein CDC25A in testicular tissue of infertile men using qPCR and immunohistochemistry, respectively. It was found that there was a decrease in mRNA expression in patients with spermatogenesis failure of several categories of Johnsen assessment. Score 3 is characterized by spermatogonia cells, where transcription of CDC25A is also found, but with small values.

In Johnson score 4, the CDC25A protein is widely expressed in primary spermatocytes. The decrease in CDC25A mRNA expression indicates an interruption in the spermatogenetic process. In Johnsen score 5, expression values are also lower than the control, probably due to the interruption of meiosis in primary spermatocytes. If the value of the expression is high, the cell division stages will continue. This suggests that CDC25A acts as an important regulator of cell cycle progression or cell proliferation.

The low expression rate of CDC25A in infertile men is the result of the reduction of spermatogenic cycles in the seminiferous tubules and shows that the CDC25 phosphatase contributes to the hyperphosphorylation of M-phase promoting factor (MPF) and CDC25 that are important to complete the meiosis (24). The low CDC25A phosphatase results in inhibition of MPF formation activity that inhibits germinal cells to enter mitosis and meiosis, which results in germ cells having arrest and degeneration occurs (25).

The low expression of mRNA is also associated with low levels of protein. This suggests that the CDC25A content in human testes is regulated at the mRNA level. The upstream factor of CDC25A is probably very important in CDC25A mRNA expression. The human “Deleted in Azoospermia” (DAZ) gene family contains at least three members represented by DAZ, DAZ-like (DAZL) and BOULE, all of its genes being expressed in germ cells. In Drosophila, the expression of Twine protein, which is a Cdc25-type phosphatase, correlates with the intracellular BOULE accumulation and is significantly reduced by BOULE mutants suggesting that BOULE can affect the expression of Twine via a direct bonding to the mRNA twine (26). In mice, Dazl binds directly to 32 UTR of the CDC25A mRNA stating that the DAZ gene family can regulate CDC25A mRNA (26). In addition, CDC25A can accumulate as a result of transcription activation of E2F-1 and c-myc and elevated levels of RNA and protein stimulate cells to enter the cell cycle. Therefore, low CDC25A expression in infertility may be caused by mutations/polymorphisms that affect the family of DAZ, E2F-1 and c-myc genes in the metabolic cascade or other factors that interfere with mRNA production or stability (25).

Previous studies of testicular biopsy samples on gene expression analyzed the relationship between the Johnsen assessment with the cAMP responsive element modulator (CREM), protamine 1 and protamine 2 genes and emphasized on stages of spermatogenesis, in particular in the sperm packaging. CREM is a transcription factor of the protamine genes that play a role in the packing of sperm replacing histones. These investigations showed a significant correlation of CREM and protamine with the Johnsen scoring (27).

The presence of protamine in spermatogenesis can cause the buffering of other genes in terms of gene silencing. The expression of protamine mRNA was pronounced in early stages of spermatogenesis resulting in low values of CDC25A gene expression, especially in Johnsen score 5. Low CDC25A expression will result in the disruption of cell division processes leading to spermatogenesis failure at a later stage. In addition, the study by Carrel et al. (8) suggests that protamine can act as a checkpoint for successful spermatogenesis.

Relative expression of the CDC25A gene using real-time quantitative PCR was obtained by amplification of cDNAs with primers designed through Primer3 (14, 15). The target gene amplicon of 87 *bp* was based on adjustment on good primer requirements in the amplification of target genes using real-time PCR with an amplicon count of 75–200 *bp* (19). Although shorter amplicons have higher amplification efficiency, the lower limit of 75 *bp* makes it easier to distinguish whether a dimer is formed by the primer (19). The primers should have an ideal length of 16–28 nucleotides and a GC content of 35% –65%. The primer used in this study was adjusted to a characteristic length of 20 nucleotides, containing 55% primary and reverse GC with a melting temperature of 56*°C*; dimer or hairpin structures were not detected (17). The primer optimization for the mRNA expression resulted in melting curves with a single peak indicating primer specificity to the target gene sequence (17, 28).

The expression of CDC25A protein was determined by calculating the number of CDC25A-positive cells; this protein is expressed in the cytoplasm. Immunohistochemical methods were performed to determine the location and distribution of proteins in spermatogenic cells in seminiferous tubules (29).

The histopathological examination of the testicular biopsy has a special value in the treatment of cases of male infertility. This assessment is primarily directed against the seminiferous tubules, both in shape and size as well as in the type and number of cells present therein. The use of the scoring method obtained several diagnoses that describe the changes or abnormalities that occur in the testicles (6, 30, 31).

According to the observations, the CDC25A protein is expressed mainly in spermatocytes. In the histological observation of testicular tissue, secondary spermatocytes are rare or not found because they have little time to enter the next cleavage. In addition to spermatocytes, the CDC25A protein is also found in spermatid cells, sperm cells and is found in spermatogonia and Sertoli cells with a weaker staining intensity than other cells (25). This explains that the CDC25A protein in spermatogonium and Sertoli cells are actively acting cells undergoing mitotic division and CDC25A is a cell cycle regulator of either mitosis or meiosis. Weak staining caused by the CDC25A protein is widely present in the primary spermatocyte cytoplasm resulting in a higher intensity staining of spermatocytes than in spermatogonium and Sertoli cells.

Lin et al. demonstrated that the CDC25A protein was found in the cytoplasm of spermatogonial cells, pachytene spermatocytes stages, early to elongated spermatids from testicular tissue samples (31). In the study by Mizoguchi and Kim, it was mentioned that the location of the CDC25A protein was expressed in the cytoplasm of spermatogonia, spermatocyte, and round spermatid cells, but not found in Sertoli cells and elongated spermatids (32).

The CDC25A protein found in cells that have not been cleaved indicates that the CDC25A function is not only related to cell cycle but also related to physiological process in spermatozoa maturation, and this mechanism is not yet known clearly. Based on the research carried out by Cheng et al., protein CDC25A is found in spermatozoa with immunocytochemical techniques and observed with a confocal microscope andit is known that the tail of spermatozoa shows a stronger color intensity than the head. The tail of the sperm is prepared by mitochondria, outer dense fiber (ODF) and axoneme. The positive results observed in the tail of sperm showed that there is a relationship between the CDC25A protein and the motility of the sperm. The presence of a positive signal of immunostaining results in the head of spermatozoa containing enzymes for the penetration of oocytes shows the role of CDC25A in the fusion of sperm with oocytes. It is also possible that the CDC25A protein is present in the cytosol of the sperm and is not related to specific organelles. The role of the CDC25A protein in motility and the interactions between sperm and oocytes requires further study (25).

The cell cycle transition in eukaryotic cells is regulated by the CDK group. This kinase activity is controlled by other proteins such as cyclin and CDK inhibitors. CDK is inactivated by the inhibition of phosphorylation of Thr-14 and Tyr-15. The inactivated cyclin/CDK complex is dephosphorylated and activated by the CDC25 phosphatase, so CDC25 phosphatase is an important regulator of cell cycle progression (33). In addition, the role of the CDC25A protein in cell division is related to MPF which consists of the catalytic subunit of cyclin B and Cdc. In addition, the role of CDC25A protein in cell division is related to MPF consisting of cyclin B and Cdc catalytic subunit. This MPF required the cells to enter the M phase, either in mitosis or meiosis. This MPF activity is controlled by the dephosphorylation of the CDC25 family and the inhibition of phosphorylation by the Wee-l protein kinase that negatively regulates the entry into mitosis by catalyzing the inhibitory tyrosine phosphorylation of the Cdc2 protein (34). According to Lin et al., MPF and its regulators are involved in spermatogenesis so that the expression of MPF and its regulators may be correlated with the testicular phenotype and the production of haploid spermatozoa (35).

Research on the relationship between mRNA expression and protein expression is generally carried out because the expression of mRNA depends on the expression of the protein, while the expression of the protein is based on the expression of mRNA. The cellular function depends on the mRNA and protein molecules and the interaction between these two components is important in the management of complex cellular functions (36).

The correlation between mRNA expression and the number of CDC25A-positive cell counts was calculated by the SPSS program. The normality test showed that the normal undistributed data were obtained so that the correlation test was performed. Based on Spearman correlation, p-value of 0.010 (p<0.05) with Spearman correlation coefficient 0.546 was obtained. This suggests that there is a moderate positive correlation between mRNA expression and many positive CDC25A protein cells in the seminiferous tubules. The Spearman correlation coefficient between 0.40–0.70 shows a moderate correlation between two variables (37).

The statistical results show a positive correlation between the relative expression of CDC25A mRNA and the percentage of positive cell counts of CDC25A protein, but when observed in each Johnsen assessment, there is a fluctuating pattern of mRNA expression, a high assessment from 3 to lower assessment 4 and 5. The relative expression returned to increase again in assessments 6 and 7. On the contrary, the pattern of protein expression shows a pattern that increases in value in each expression.

Some studies explain that mRNA levels of a gene cannot always predict protein levels. Schwanhausser et al. (38) performed an analysis of mouse fibroblasts and found that abundance of housekeeping gene encoding ribosomal proteins, glycolytic proteins, and Krebs cycle proteins tended to have stability at mRNA and protein levels. In contrast, proteins associated with transcription factors, signal transduction, chromatin modification and cell cycle functions tendency have unstable mRNAs and proteins. The most important regulators in cellular division and differentiation are considered to have a weak correlation between mRNA and protein levels (38).

According to Vogel et al. (39), transcription, mRNA damage, translation, and protein degradation are key processes that determine the stability of protein concentrations. In addition, other factors, like microRNA (miRNA) affect the translation process. It is known that miRNA simultaneously suppresses hundreds of genes by inhibiting the translation of mRNA into protein (40). Post-translational mechanisms also control protein abundance, *e.g*., hundreds of ubiquitin ligases, proteases, tyrosine and serine-threonine kinases and phosphatases in the human genome that affect the abundance of protein levels; among them, phosphorylases regulate the mechanisms that control cell division and differentiation (41).

Gene expression mostly describes the activity of transcription and does not always correlate with the expression of the protein. This is due to post-transcriptional and post-translational processing that influences the biological protein activity (42). Referring to this study, although the expression of CDC25A mRNA may increase, not necessarily all transcripts are translated and processed to form functional CDC25A protein.

## Conclusion

Johnsen scoring 5 exerts highly significant correlation with CDC25A-positive cells indicating CDC 25A protein expression, which in turn correlates with mRNA expression. Decreased CDC25A expression in Johnsen score 5 is associated with meiotic arrest in the etiology of spermatogenic failure. Further studies should clarify how the stimulation and stabilization of CDC25A mRNA and protein expression, *e.g*., via the influence of protamine can improve spermatogenesis in infertile men.
